# UNHEALTHY COPING: SMOKING AND DRINKING BEHAVIORS AND RELATED RISK FACTORS AMONG DEMENTIA CAREGIVERS

**DOI:** 10.1093/geroni/igae098.0324

**Published:** 2024-12-31

**Authors:** Jiaming Liang, Maria Aranda

**Affiliations:** University of Texas Health Science Center at Houston, SAN ANTONIO, Texas, United States; University of Southern California, Los Angeles, California, United States

## Abstract

Dementia caregivers may adopt unhealthy lifestyle behaviors as a form of stress relief. While some interventions promoting healthier behaviors have been developed for dementia caregivers, their smoking and drinking behaviors have received limited attention. This study aimed to explore (1) the disparities in smoking and drinking behaviors between dementia caregivers and non-caregivers, and (2) potential risk factors associated with these behaviors among dementia caregivers. Utilizing data from the 2017 Behavioral Risk Factor Surveillance System, we conducted cross-sectional analyses on 280 dementia caregivers and 9,878 non-caregivers. Multinomial regression revealed that dementia caregivers are more likely to be daily e-cigarette users compared to never-users (RRR = 0.33, p = 0.04). Additionally, zero-inflated negative binomial regression indicated that dementia caregivers tend to consume 0.22 more alcoholic drinks per occasion than non-caregivers (p = 0.03). Interaction analyses demonstrated that younger age and lower income are associated with daily tobacco use. In contrast, factors such as older age, being unmarried, home ownership, higher income, employment, fewer chronic conditions, and absence of depression are related to increased alcohol consumption among dementia caregivers (i.e., drinking on more days in the past 30 days, and consuming more drinks per occasion). This study underscores the increased risk of unhealthy smoking and drinking behaviors among dementia caregivers and highlights the need for targeted interventions to address these risk factors and promote healthier lifestyle choices among this population.

